# A Comparative Study on the Functional Response of *Wolbachia*-Infected and Uninfected Forms of the Parasitoid Wasp *Trichogramma brassicae*

**DOI:** 10.1673/031.010.14127

**Published:** 2010-10-01

**Authors:** S. Farrokhi, A. Ashouri, J. Shirazi, H. Allahvari, M.E. Huigens

**Affiliations:** ^1^ Biological Control Research Department, Iranian Research Institute of Plant Protection, P. O. Box, 1454, Tehran 19395, Iran; ^2^ Department of Plant Protection, College of Agriculture, University of Tehran, Karaj 31587–11167, Iran; ^3^ Laboratory of Entomology, Wageningen University, P.O. Box, 8031, 6700 EH, Wageningen, The Netherlands

**Keywords:** egg parasitoid, endosymbiotic bacterium, attack rate, handling time, thelytokous strain, biological control, *Sitotroga cerealella*

## Abstract

*Trichogramma* species (Hymenoptera: Trichogrammatidae) are haplo-diploid egg parasitoids that are frequently used as biological control agents against lepidopteran pests. These wasps display two reproductive modes, including arrhenotoky (bisexuality) and thelytoky (unisexuality). Thelytokous forms are often associated with the presence of endosymbiotic *Wolbachia* bacteria. The use of thelytokous wasps has long been considered as a way to enhance the efficacy of biological control. The present study investigates the potential of a thelytokous *Wolbachia*-infected and an arrhenotokous uninfected *Trichogramma brassicae* Bezdenko strain as inundative biocontrol agents by evaluating their functional response towards different egg densities of the factitious host, the Angoumois grain moth, *Sitotroga cerealella* (Olivier) (Lepidoptera: Gelechiidae). The results revealed a type II functional response for both strains in which parasitism efficiency decreases with host egg density because of an increasing host handling time. A model with an indicator variable was used to compare the parameters of Holling's disc equation in different data sets. It was demonstrated that the two strains did not differ in host attack rate. However, the *Wolbachia*-infected strain did have an increased host handling time when compared to the bisexual strain. Some applied aspects of the findings are discussed.

## Introduction

*Trichogramma* species are well-known polyphagous egg parasitoids that have been used extensively in inundative biological control programs against lepidopteran pests in different agroecosystems (Li 1994; Smith 1996; van Lenteren 2000). These wasps are haplo-diploid and typically produce male offspring from unfertilized eggs and female offspring from fertilized eggs (arrhenotoky). However, certain strains of *Trichogramma* include thelytokous females that produce only female offspring, even from unfertilized eggs. Out of about 180 species described (Pinto 1998); at least 18 species contain thelytokous forms (Pinto and Stouthamer 1994; Stouthamer 1997; Almeida 2004; Farrokhi 2010). In most cases, thelytokous *Trichogramma* wasps are infected with parthenogenesis-inducing (PI) *Wolbachia* (Stouthamer et al. 1993). Endosymbiotic *Wolbachia* bacteria are known to affect the fecundity and dispersion of infected strains (Stouthamer and Luck 1993; Silva 1999). In some cases infection with PI-*Wolbachia* also have severe negative effects on competitive ability under conditions of superparasitism and on the survival of immature stages (Tagami et al. 2001; Hohmann et al. 2001; Huigens et al. 2004; Miura and Tagami 2004). Additional effects on other important biological control traits such as host searching ability may also be expected. Nevertheless, thelytokous strains of *Trichogramma* may be superior biological control agents under conditions of host limitation (Stouthamer and Luck 1993; Stouthamer 1993; Silva et al. 2000). In theory, the advantages of the use of thelytokous parasitoid wasps are: 1) their high rate of increase, 2) their inexpensive production as all wasps are female, 3) their easy establishment because they do not require finding a mate, and, therefore, 4) their effectiveness at low host densities (Stouthamer 1993).

Efforts have been made to find the best species or strain of *Trichogramma* for control of a particular pest (Hassan 1988). The success of such programs has been variable, and attention has been focused on selection of the most effective species and strains of *Trichogramma* (Hassan 1990; Smith 1996). In this regard, several biological characteristics such as searching ability, fecundity, longevity and sex ratio have been used to assess potential efficacy of a parasitoid. Above all, searching speed has been adopted as a quality measure for mass-produced *T. brassicae* wasps (Bigler 1989; Cerutti and Bigler 1995), because of its theoretical link to host finding (parasitoids that move faster should find more hosts) and its correlation with parasitism in the field (Bigler et al. 1988). The searching and walking speeds of thelytokous females of *T. minutum* were significantly higher compared to arrhenotokous conspecifics (van Hezewijk et al. 2000). The effects of *Wolbachia* on *T. cordubensis* and *T. deion* under laboratory and greenhouse conditions were also evaluated (Silva et al. 2000). Arrhenotokous females dispersed more in the laboratory. However, in the greenhouse, thelytokous lines showed a higher potential for biological control than their arrhenotokous conspecifics. Another study indicated that *Wolbachia*-infection did not affect the walking activity and other behavioral components of *T. atopovirilia* Oatman & Plainer (Almeida 2004).

Other important aspect in evaluating the efficiency of a natural enemy is the attack rate across a range of host densities, i.e., its functional response (Berryman 1999). Holling (1959, 1966) proposed three types of functional response. He modeled type II using the “Disc Equation”:

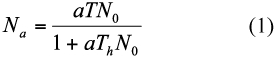

where *N_a_* is the number of host attacked, *a* the attack rate, which relates encounter rate with host to *N*_0_ (the initial host density), *T* the total available searching time and *T_h_* the handling time per host.

The type of functional response of *Trichogramma* species has generally been found to be either type I or type II (Smith 1996). However, a type III response has also been reported (Wang and Ferro 1998). The parasitism efficiency in a type II functional response decreases as the total handling time increases with host egg density, whereas type III is generally related to an increase in searching activity when host densities increase at low, but not at high, host densities (Hassel 1978). Different abiotic and biotic factors may influence the functional response, such as temperature and prey or host species (Wang and Ferro 1998; Mohaghegh et al. 2001; Allahyari et al. 2004; Kalyebi et al. 2005; Reay-Jones et al. 2006; Moezipour et al. 2008). Differences in functional response among species and strains may also be caused by genetic and/or phenotypic differences. Here, a study was done to compare the functional response of an arrhenotokous and a *Wolbachia*-infected thelytokous strain of *Trichogramma brassicae* Bezdenko. *T. brassicae* is a widely used biological control agent against different pest species. In Iran, it is the dominant *Trichogramma* species and has been reared and released for biological control of some local key pests such as the rice stem borer *Chilo suppressalis* (Walker), the European corn stem borer *Ostrinia nubilalis* (Hübner) and the carob moth *Ectomyelois ceratoniae* (Zeller) (Ebrahimi et al. 1998). Here, the functional response of *Wolbachia*-infected and uninfected *T. brassicae* was evaluated on eggs of the factitious host, the Angoumois grain moth, *Sitotroga cerealella* (Olivier) (Lepidoptera: Gelechiidae). Eggs of this species are extensively used in mass production of *Trichogramma* species worldwide (Greenberg et al. 1996).

## Material and Methods

### Parasitoid cultures

In 2005, a survey in Baboulsar (South East of the Caspian Sea, Iran) resulted in the collection of parasitized *Ostrinia nubilalis* (Hübner) egg batches laid on *Xanthium strumarium* L. (Asterales: Asteraceae). Emerging *Trichogramma* wasps were reared separately under laboratory conditions in the Biological Control Research Department (BCRD) of the Iranian Research Institute of Plant Protection (IRIPP). Afterwards, the species was identified as *T. brassicae*, based on genitalia shape at IRIPP and the size and sequence of the rDNA internal transcribed spacer 2 (ITS-2 region) (Ebrahimi et al. 1998; Stouthamer et al. 1999) at the Laboratory of Entomology of Wageningen University as described in Gonçalves et al. (2006). Voucher specimens were kept at BCRD. Specimens with highly female biased sex ratio were checked for the presence of PI-*Wolbachia* using PCR with specific primers for the *wsp* gene (81F/691R primers; Braig et al. 1998). Two separate isofemale *Wolbachia*-infected and uninfected *T. brassicae* strains were established in glass vials (35 × 200 mm) on eggs of the Angoumois grain moth (*S. cerealella*) in a growth chamber at 20 ± 1° C, 55 ± 20% RH and 16:8 L:D photoperiod. The arrhenotokous and thelytokous (*Wolbachia*-infected) strains were designated as B and BW^+^, respectively. The GenBank accession numbers FJ441291 and FJ441292 were allocated to the *wsp* and ITS-2 sequence of the BW^+^-strain. At the time of the experiment, *Trichogramma* wasps had been reared for 25 generations.

### Functional response experiment

To determine the functional response of the strains, eight host densities (2, 5, 10, 20, 30, 40, 60 and 80) of fresh *S. cerealella* eggs were prepared by randomized dispersion of eggs on a 10 × 35 mm strip of white card. Twenty newly emerged females (and mated in case of the B strain) of either strain were confined individually in glass vials (16 × 100 mm) and provided with an egg card of defined density and a fine streak of diluted honey as food. All prepared vials (2 strains × 20 individuals × 8 densities = 320) were kept at 25 ± 1° C, 60 ± 15% RH and 16:8 L:D. After 24 h, the wasps were removed from the vials and eggs were left at the same environmental conditions until they turned black. The blackened (parasitized) eggs were counted and recorded by strain and density. The dimension of the length of the ovipositor of both wasp strains was measured as a size index (Grenier et al. 2001).

### Data analysis

Analysis of functional responses comprised of two distinct steps (Messina and Hanks 1998; De Clercq et al. 2000; Juliano 2001; Mohaghegh et al. 2001; Allahyari et al. 2004). In the first step, the curve shape or type of functional response was determined, typically by determining if the data fit a type II or III functional response. For this purpose, logistic regression of the proportion of parasitized hosts (*N_a_*) vs. the initial number of hosts (*N_0_*) is the most effective way. Therefore a polynomial function was fitted as follows (Juliano 2001):

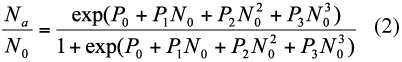

where *P*_0_, *P*_1_, *P*_2_ and *P*_3_ are the parameters to be estimated. These parameters were estimated using the CATMOD procedure in SAS software (see Juliano 2001). The two data sets were individually fit to the model (2) and the type of functional response was determined. Significant negative or positive linear coefficients in the expression were fit by the method of maximum likelihood to data on proportion of *N_a_* / *N_0_* indicate type II and type III functional responses, respectively. The sign of *P*_1_ and *P*_2_ was used to distinguish the shape of the curves. A positive linear parameter (*P*_1_) and a negative quadratic parameter (*P*_2_) indicate that functional response is type III, whereas the functional response is type II when both parameters are negative. In the second step, a nonlinear least square regression was used (NLIN procedure with DUD method in SAS) to estimate the functional response parameters of the Holling's disc equation. Then, the obtained parameters were compared [*T_h_*, and either *a* (for type II) or *b, c*, and *d* (for type III)]. Comparison between the two functions was performed using an equation with indicator variables:

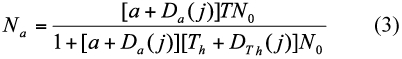

where *j* is an indicator variable that takes value 0 for B-strain and 1 for BW^+^-strain. The parameters *D_a_* and *D_Th_* estimate the differences between the strains in the value of the parameter *a* and *T_h_*, respectively. In other words, the handling time for the B-strain is *T_h_*, and for the BW^+^-strain is *T_h_* + *D_Th_.*- To find a difference between the two handling times (for B and BW^+^), it must be proved that *D_Th_* is a significant number, and it is not equal to zero. If *D_Th_* is not significantly different from zero, the difference between *T_h_* and *T_h_*, + *D_Th_* is not significant and the two handling times are not statistically different (Juliano 2001). The coefficient of determination was calculated as r^2^ = 1 — residual sum of squares/corrected total sum of squares.

**Table 1.**
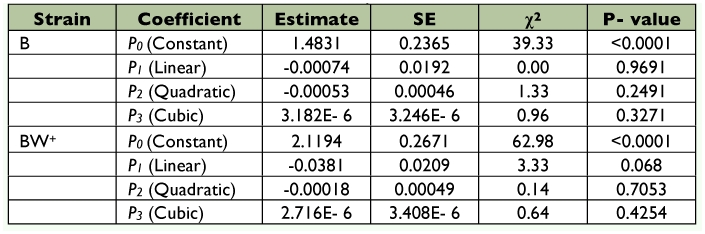
Maximum likelihood estimates from logistic regression of the proportion of *Sitotroga cerealella* eggs parasitized by two strains of *Trichogramma brassicae* as a function of initial host density.

**Figure 1.**
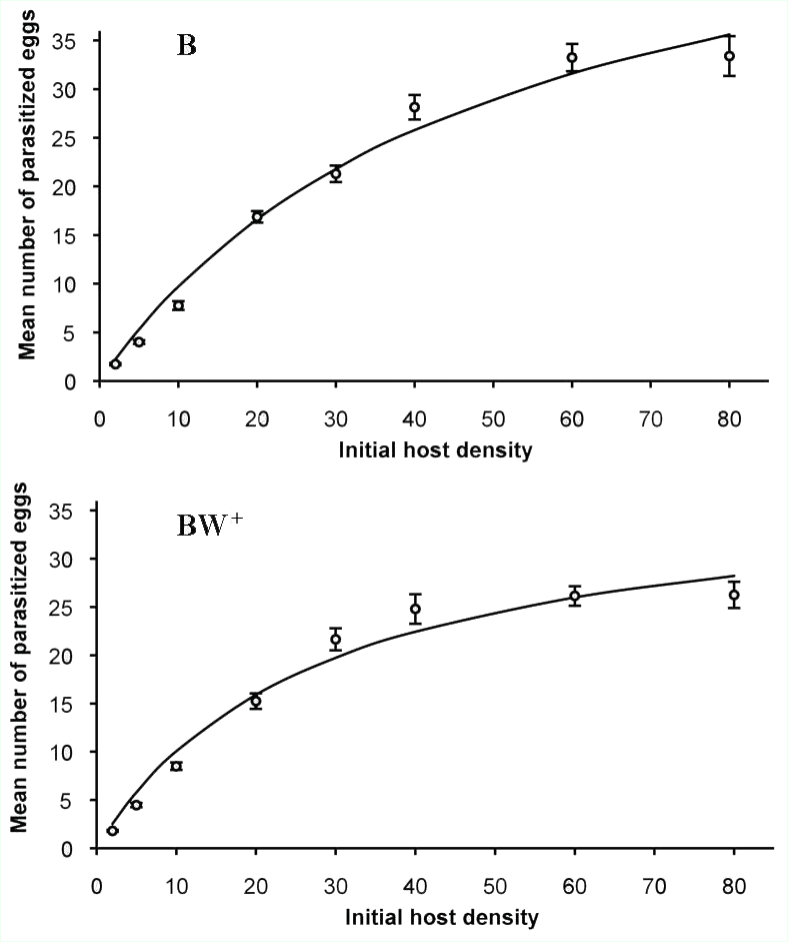
Functional response of B (above) and BW^+^ (below) strains of *Trichogramma brassicae* to eight different initial densities of *Sitotroga cerealella* eggs. Symbols: observed mean ± SE. The lines are the predicted responses from the model. High quality figures are available online.

## Results

The outcome of the logistic regression indicated a type II functional response for both strains of *T. brassicae* as the sign of the linear term was negative in both cases (Table 1). Functional response curves of female adults of the strains to various densities of host eggs are shown in Figure 1. Estimated *a* values and host handling times for B and BW^+^ strains were 0.0487 h^-1^ and 0.417 h, and 0.0569 h^-1^ (0.0487+0.0082) and 0.6307 h (0.417+0.2137), respectively. The maximum number of attacks is limited by an upper asymptote value defined by the ratio of *T/T_h_* (Hassel 1978). Theoretically, the maximum parasitism rate by the B- and BW^+^-strains was 57.55 and 38.05 host eggs per day, respectively. As shown in Table 2, the asymptotic 95% confidence interval for *D_b_* included 0 but that of *D_Th_* was greater than 0, showing that there was a significant difference between *T_h_* and *T_h_* + *D_Th_*. Obviously, the two populations have a functional type II response with a significant difference in host handling time but similar attack rate. The coefficients of determination (r^2^) indicated equal variation in parasitism rates of *T. brassicae* strains (Table 2). Ovipositor lengths differed significantly between the two *T. brassicae* strains (*p* = 0.0004, t-test with α = 0.01), with mean values (± SE) of 0.1645 mm (± 0.00126) for the B-strain and 0.1581 mm (± 0.00115) for the BW^+^-strain.

## Discussion

Our study showed that the *Wolbachia*-infected and uninfected *T. brassicae* strains had only a slight difference in their functional type II response. Functional response studies are useful in providing the first step for comparing the efficiency of different species/strains (Overholt and Smith 1990) and also provide information on host-finding abilities of candidate natural enemies (Munyaneza and Obrycki 1997). The exactness of functional response as a comparison tool is highly related to the use of appropriate models and data analysis; the use of inappropriate models and analysis methods may result in an incorrect estimation. Holling's equation can be used only when Rogers' model does not enable the researcher to estimate valid parameters. For example, Holling's model has previously been used because Rogers' model provided invalid parameters (Mohaghegh et al. 2001; Allahyari et al. 2004). As re-encounter occurred in our experiment, the random parasite equation was used first but this model did not help to estimate appropriate parameters (*a* and *T_h_* were negative). For this reason, the Holling's disc equation was used.

**Table 2.**
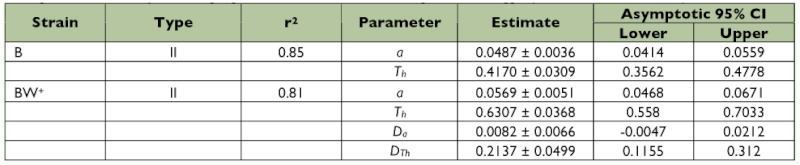
The r^2^-value and parameters (mean ± SE) estimated by the disc equation with indicator variable (3) for two strains of *Trichogramma brassicae* parasitizing eight different densities of *Sitotroga cerealella* eggs, (CI = confidence interval).

Similar to most previous reports on *Trichogramma* under laboratory conditions, a type II functional response was obtained for the both strains of *T. brassicae*. However, a type I response to host densities has been reported for *T. minutum* with *Ephestia kuehniella* eggs (Mills and Lacan 2004). Moreover, a type II functional response was found with *T. ostriniae* parasitizing *O. nubilalis* at low temperatures and a type III at high temperatures (Wang and Ferro 1998). Some studies on Iranian strains of *T. brassicae* indicated a type II response with *S. cerealella* at 25° C (Karimian 1998; Moezipour et al. 2008). The type of functional response and estimated parameters for an insect species could be affected by some factors such as host plant, temperature and type of prey or host (Juliano and Williams 1985; Coll and Ridgway 1995; Runjie et al. 1996; Messina and Hanks 1998; Wang and Ferro 1998; De Clercq et al. 2000; Mohaghegh et al. 2001; Moezipour et al. 2008). However, there has been no study on the effect of *Wolbachia* on the functional response of parasitoids/predators.

The estimated host handling time of the BW^+^strain in this study (0.6307 h) is significantly longer compared with that of the B-strain (0.4170 h). In other words, the thelytokous *Wolbachia*-infected wasps spent more time handling the host. However, the biological basis of this difference remains unknown. Handling time is a general term that includes time for finding, drumming and parasitizing a host, time for resting, preening and sap feeding in parasitoids. The difference in host handling time may be due to the difference in size between the BW^+^- and B strain. Size has been shown to be positively correlated with host fitness in *Anagyrus kamali*, a mealybug parasitoid (Sagarra et al. 2001). In addition, the host handling time of larger *Trissolcus grandis* wasps emerging from more suitable host eggs, has been shown to be shorter than that of smaller conspecifics (Allahyari et al. 2004). Therefore, a higher *T_h_* in the BW^+^strain may be a result of a reduced body size (e.g., a smaller ovipositor length as a body size index) when compared to the B strain.

In conclusion, infection with *Wolbachia* does not seem to have a significant impact on the type of functional response and attack rate of the studied parasitoids under controlled laboratory conditions. However, the infected strain of *T. brassicae* had a lower quality in comparison to the B-strain with respect to the host handling time and magnitude of parasitism capacity. To confirm whether this difference is caused by *Wolbachia*, future studies should include more strains and also test infected strains that are transformed into arrhenotokous uninfected strains through antibiotic treatment against original infected strains that did not receive antibiotics (Silva et al. 2000). One should be careful when extrapolating the results on functional responses under laboratory conditions as described in the present study (using small vials and a single host species) to parasitoid behavior under natural conditions. In our laboratory study, the *Wolbachia*-infected and uninfected *T. brassicae* strain parasitized equally well under low host densities because they had ample time to search for host eggs in a small vial. In contrast, *Wolbachia*-infected wasps may perform better under low host densities in the field when host eggs are distributed over a large spatial scale (Stouthamer 1993). The results obtained in this study may be useful for the evaluation of unisexual *Wolbachia*-infected *T. brassicae* as biological control agents of lepidopteran pests such as the rice and corn stem borers. Both pests lay their eggs in batches. Infected wasps showed an increased host handling time compared to uninfected wasps but handling time may be of less importance for parasitoids attacking hosts in clumps.

## Abbreviations

a: attack rate or instantaneous search rate;
B: *Wolbachia*-uninfected strain collected from Baboulsar;
BCRD: Biological Control Research Department;
**BW**^+^: *Wolbachia*-infected strain collected from Baboulsar;
IRIPP: Iranian Research Institute of Plant Protection;
ITS-2: internal transcribed spacer 2 region;
*N_a_*: number of parasitized hosts;
*N_o_*: initial host density;
Pl: parthenogenesis inducing;
*T*: total available searching time;
*T_h_*: handling time per host;
*wsp*: *Wolbachia* surface protein gene
